# Infantile Dentition

**Published:** 1857-01

**Authors:** 


### SELECTED ARTICLES.

### ARTICLE VI.

### *Infantile Dentition.*

*Morbid Dentition.*—In our preceding communication on Infantile Dentition, we directed attention to the minor ailments during teething, and remarked that careful hygienic management was the only means necessary to bring such cases to a successful termination. We shall now proceed to the consideration of the pathology and treatment of the severer and more dangerous disorders that frequently supervene, during the eruption of the temporary teeth, and which, with the exception of contagious and epidemic maladies, comprise the greater part of the diseases of infantile life.

The symptoms of morbid dentition are referable to inflammation or induration of the gums, and to the various sympathetic and constitutional disorders which originate in these sources of local irritation. When the gums are inflamed, they become hot, dry, and injected, and so exquisitely tender, that the infant often refuses to seize the nipple. General symptoms are speedily superadded, and are marked by heat of skin, quick pulse, vitiated excretions, hurried respiration, and disturbed sleep. The secretion of saliva is sometimes abundant, at other times it is remarkably deficient. The inflammation occasionally extends to the salivary glands, and to the mucous membrane of the lips or cheeks; and it not unfrequently happens that the gums become ulcerated.

When the gums are merely indurated, there is very little tenderness, no redness of the part affected, and the secretion of saliva is not increased. The gum is thickened, but, otherwise, presents an appearance so natural, that this lesion, which is of the greatest importance, is frequently overlooked.

The principal thing in the treatment of these cases, is to lance the gums freely. A superficial incision will be of no avail; the gums must be cut down until the lancet impinges on the approaching tooth. The only caution required, is that the incision should be inclined outwards, in order to avoid the tissues which connect the permanent with the temporary teeth. The shape of the gum lancet is a matter of consequence. In order to keep the instrument steadily fixed in the handle, it should have a spring back; the blade should be rather round and broad, and extremely sharp. The operation requires considerable skill and caution to ensure its safe and effectual performance. The terrors of the mother and the restlessness of the infant, frequently render it by no means an easy operation; and the careless operator is apt to wound either the cheeks or tongue, to make the incisions too superficially to be of the slightest vse.

The prejudices of former writers against this invaluable operation scarcely require comment; but as we still find a few, and we are happy to say a very few individuals, who retain a bigoted faith in the absurd dogmata of their forefathers, we will briefly refer to the objections which have been urged against the utility of lancing the gums. The first is, that the cicatrix, which is formed after the division of the gum, is likely to offer more resistance to the passage of the tooth than the natural tissues of the part. This notion is entirely opposed to the truth; the preternatural readiness with which recent structures are absorbed, is shown in the liability of newly formed cicatrices or wounds of the extremities to become ulcerated. But were it otherwise, no injury would result from a repetition of the operation, a mode of proceeding which is often required when the first division of the gum has failed to give relief. The second objection which has been advanced, is the danger of serious hemorrhage from the gums. The possibility of such an accident occurring once in a hundred-thousand cases, in consequence of a hemorrhagic diathesis, constitutes a contingency so remote, that we need not burthen the memory with a recollection of it. One other charge against the propriety of the operation remains to be disposed of. It has been urged, that there is danger of injuring the second set of teeth, which are placed under the milk-teeth. Nothing short of the clumsiest manipulution could effect a serious injury of this description. By exercising the precautions which we have previously recommended, such a result is impossible.

In addition to free division of the gums, it is advisable in cases of severe dentition, that the bowels should be well opened; if, however, the action of a purgative should be followed by a tendency to hypercatharsis, with tormina and copious mucous stools, it will be necessary to check these symptoms by the exhibition of chalk mixture and ammonia. It must however, always be borne in mind, that brisk purgation is one of the most effectual means of counteracting the local irritation of teething.

The most important of the general symptoms are those occasioned by the *reflection* of the irritation to the brain and nervous system. When this condition obtains, the pupils of the eyes are dilated; the child moans continually, and waves its head in a distressing and irritable manner. Violent convulsions often ensue, and recur in quick succession, until death, provided prompt relief is not procured, puts a period to the infant's sufferings. An extensive practice connected with affections of this class, has convinced us that the intimate connection of cerebral diseases with infantile dentition has not been sufficiently recognised, and that a more general acquaintance with the fact would be the means of saving many an infant from a premature grave.

A very common effect of teething is diarrhea, attended with tormina and tenesmus. When the purging is not excessive, as we have previously observed, it ought not to be arrested. The infant will emaciate under its influence; but as soon as the teeth appear, the child will begin to regain its flesh and strength. Vomiting is an occasional complication with diarrhea, and may require for its removal a mustard poultice or a blister to the epigastrium.

Eruptive affections of the skin are amongst the commonest and most troublesome sequellæ of teething. Papular and vesicular eruptions make their appearance, especially on the head and face, and are often extremely difficult of removal. Their appearance is often followed by a subsidence of the constitutional disturbance, and on this account there is naturally a popular prejudice against arresting what is supposed to be a salutary effort of nature. The lower classes are so strongly imbued with this notion that they allow a moist form of eczema, which often attacks the ears, to run riot as it were, until the parts become violently inflamed and excoriated. Although it would be highly imprudent to arrest this affection hastily, nevertheless, after the teeth have cut the gums, the disease should be remedied by means of aperients, and a lotion of sulphate of zinc.

Spasm of the glottis is another symptom of an alarming kind, which is occasionally produced by the irritation of morbid dentition. It is marked by a loud crowing sound resembling that of croup, but differing from the latter disease in being purely of a spasmodic character, and from the momentary duration of the paroxysms. The attacks generally occur on the infant's awakening from sleep, or on its swallowing food. When the paroxysms are severe, the danger of suffocation is imminent; the face and eyes become intensely suffused, the eyeballs protrude, and the general appearance is that of agony from suffocation. After a few moments the spasm of the glottis subsides, followed by the characteristic inspiratory noise. In some few instances, the face although indicative of intense suffering, continues pale during the attack. After all that we have observed on the importance of scarifying the gums, we need not insist on the paramount necessity of having immediate recourse to the operation in this alarming affection. Its beneficial effects are often immediate; as, however, the paroxysm is apt to recur on the eruption of every fresh tooth, it will be expedient to have recourse to precautionary measures. Laxatives and warm baths should be administered daily; a blister should also be applied behind each ear; and if the child resides in a crowded town or metropolis, change of air is indispensable for its recovery.

We have now enumerated the principal sympathetic disorders consequent on morbid dentition. Before concluding this division of the subject, it is necessary to observe that there are many intractable infantile ailments which are augmented by dentition, although they do not owe their origin to abnormal teething. This fact shows the necessity of careful attention to the gums in *every* disease of early life.

In our next communication we shall offer some remarks on caries and necrosis of the milk teeth.—*British Jour. Dent. Sci.*

